# Development of multiplex PCR for rapid detection of metallo-β-lactamase genes in clinical isolates of *Acinetobacter baumannii*

**Published:** 2020-04

**Authors:** Reza Ranjbar, Shahin Zayeri, Amir Mirzaie

**Affiliations:** Molecular Biology Research Center, Systems Biology and Poisonings Institute, Baqiyatallah University of Medical Sciences, Tehran, Iran

**Keywords:** *Acinetobacter baumannii*, Metallo-β-lactamase, Multiplex polymerase chain reaction

## Abstract

**Background and Objectives:**

*Acinetobacter baumannii* has been known as a major pathogen causing nosocomial infections. The aim of this study was to develop multiplex PCR for rapid and simultaneous detection of metallo-β-lactamase (MBL) genes in clinical isolates of *A. baumannii*.

**Materials and Methods:**

In this study, we used three sets of primers to amplify the MBL genes including *bla*_ OXA-48_ , *bla*_ OXA-23_ and *bla*_ NDM_ . The multiplex PCR assay was optimized for rapid and simultaneous detection of MBL genes in *A. baumannii* strains recovered from clinical samples.

**Results:**

*A. baumannii* strains recovered from clinical samples were subjected to the study. The multiplex PCR produced 3 bands of 501 bp for *bla*_ OXA-23_ , 744 bp for *bla*_ OXA-48_ and 623 bp for *bla*_ NDM_ genes. In addition to, no any cross-reactivity was observed in multiplex PCR.

**Conclusion:**

Based on obtained data, the multiplex PCR had a good specificity without any cross reactivity and it appears that the multiplex PCR is reliable assay for simultaneous detection of MBL genes in *A. baumannii* strains.

## INTRODUCTION

*Acinetobacter baumannii* is an aerobic Gram-negative bacterium that plays a major role in nosocomial infections (1, 2). Urinary tract infection, pneumonia, septicemia, wound infection and meningitis are caused by *A. baumannii* strains in patients (3, 4). In recent years, failure in treatment of this bacterium make this pathogen an important health problem (5). Plasmid mediated metallo-β-lactamase (MBL) are responsible for resistance to carbapenems (6). Carbapenem resistant *A. baumannii* strains are reported worldwide and strains with this phenotype are resistant to all classes of antimicrobial agents (7). Carbapenem resistance in *A. baumannii* strains is mediated by different mechanisms including efflux pump, reduced permeability and especially by production of oxacillinase (OXAs) and metallo-β-lactamase (8, 9). OXA-23 and OXA-48 carbapenemase producing isolates are common strains worldwide (10). MBLs-encoding genes are located on integrons that can be transferred between bacterial species using horizontal gene transfer (11).

There are four types of MBL enzymes that have been detected in *A. baumannii* strains including Imipenemase (IMP), Verona Imipenemase (VIM), Seoul Imipenemase (SIM) and New Delhi Metallo beta-lactamase (NDM)-1-types (12, 13). The NDM-1 is one of the latest and main resistance mechanisms in Gram-negative bacteria (14). Initially, the *bla*_ NDM-1_ gene was identified in *Klebsiella pneumoniae* and *Escherichia coli* in India and Pakistan and after that in other Enterobacteriaceae (15). In addition to, NDM producers *A. baumannii* strains are increasingly being identified worldwide (16). Detection of NDM producers in Enterobacteriaceae is simple but detection of *A. baumannii* producers may be much more difficult (17). The MBLs producers *A. baumannii* strains widespread in the India with 70–90% prevalence whereas in Pakistan 27.1% of strains may carry MBL genes (18).

In this study, rapid and simultaneously detection of MBLs genes including *bla*_ OXA-48_ , *bla*_ OXA-23_ and *bla*_ NDM_ were investigated by multiplex PCR.

## MATERIALS AND METHODS

### Target genes and primers.

In this study, we used three sets of primers (7) to amplify the MBLs genes including *bla*_ OXA-48_ , *bla*_ OXA-23_ and *bla*_ NDM_ . The primers sequences are shown in Table 1. In order to select the desired primers, the sequences of genes that encode carbapenemases (*bla*_ OXA-48_ , *bla*_ OXA-23_ and *bla*_ NDM _) were obtained from the GenBank databases and were aligned using MEGA 5 to identify highly homologous regions suitable for selected primers. Subsequently, three sets of primers were tested against control standard strains, as well as clinical isolates, in a single PCR reaction and then in a multiplex format. The control strains which used in this study included *Escherichia coli* ATCC BAA-2523 for *bla*_ OXA-48_ , *K. pneumoniae* ATCC strain BAA-2146 for *bla*_ NDM-1_ and *A. baumannii* NCTC 13304 for *bla*_ OXA-23_ like.

**Table 1.** Primer sequences used in this study

**Primers**	**Sequence (5′→3′)**	**Amplified fragment (bp)**	**Annealing temperature**
OXA-48	F: 5′-TTGGTGGCATCGATTATCGG-3′ R: 5′- GAGCACTTCTTTTGTGATGGC-3′	744	57
OXA-23	F: 5′-GATCGGATTGGAGAACCAGA-3′ R: 5′-ATTTCTGACCGCATTTCCA-3′	501	57
NDM	F: 5′-GGTTTGGCGATCTGGTTTTC-3′ R: 5′- CGGAATGGCTCATCACGATC-3′	623	57

### Bacterial DNA extraction.

*A. baumannii* strains were recovered from patients admitted at several hospitals including Children Medical Center and Baqiyatallah hospitals in Tehran, Iran, from January to June 2017. The CDT (Combined disk test) was used for the phenotypic detection of MBLs in *A. baumannii* strains that were resistant to carbapenems (19). Subsequently, the bacterial isolates were cultured on LB broth at 37 °C for 24 h and were centrifuged. The bacterial pellets were used as template for DNA extraction using kit according to the manufacturer’s instruction (Roche, Germany).

### Multiplex PCR for detection of metallo-β-lactamase genes.

The multiplex PCR assay was done using a total volume of 25 μl containing 1 mM MgCl_2_, 1× PCR buffer, 2 U Taq DNA polymerase, 1 pico-mole of each primers, 200 μM dNPTs and 2 μl of DNA template. The multiplex PCR condition was 30 cycles following a pre-denaturation step at 95 °C 3 min and each cycle consisted of denaturation at 95 °C for 20 s, annealing at 57 °C for 1 min, extension at 72 °C for 1 min. After 30 cycles, the final extension was 7 min at 72 °C. Finally, the amplified DNA was determined using 1% agarose gel electrophoresis and visualized by UV transluminator. In addition, for optimization of multiplex-PCR, we evaluated the primers separately in control strains in simplex PCR and then the multiplex-PCR was done.

## RESULTS

**Fig. 1.**
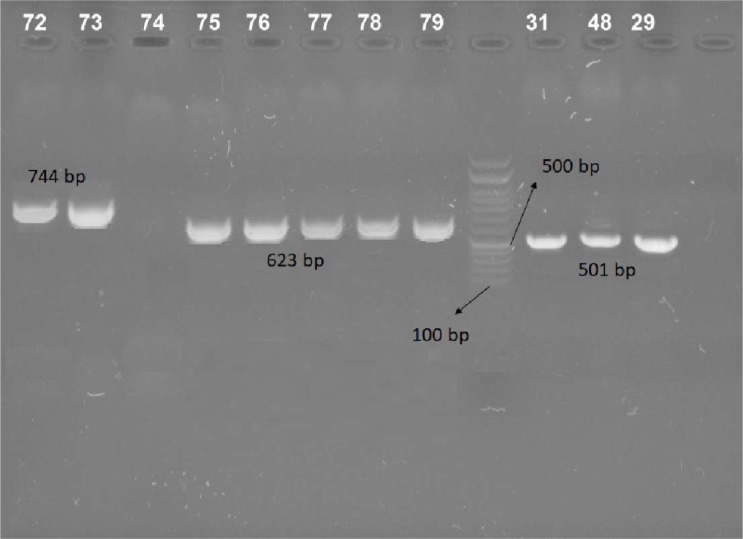
PCR amplification of *bla*_ OXA23_ (744 bp), *bla*_ NDM_ (623 bp) and *bla*_ OXA48_ (501 bp) genes in standard strains.

Standard and clinical strains of *A. baumannii* isolates subjected to the study and the PCR yielded expected bands of amplified target genes in standard strains (Fig. 1).

**Fig. 2.**
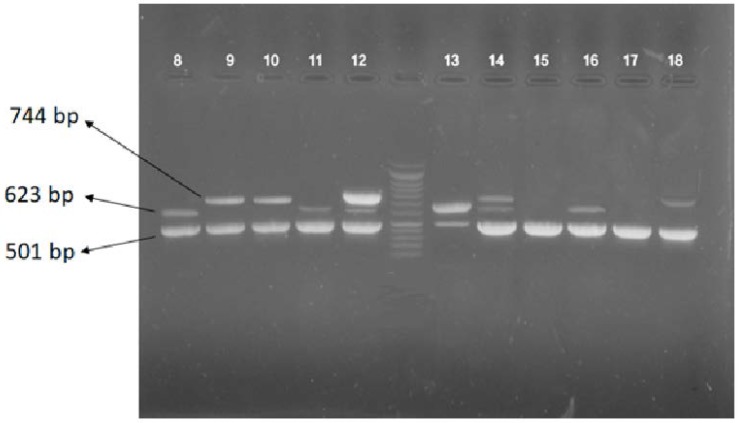
Multiplex PCR for detection MBLs genes in *A. baumannii* strains: lane 8: positive samples for *bla*_ OXA-23_ and *bla*_ NDM_ genes. Lane 9: positive samples for *bla*_ OXA-23_ and *bla*_ OXA-48_ , lane 12: positive samples for *bla*_ OXA23_ , *bla*_ NDM_ and *bla*_ OXA 48_ genes.

In multiplex PCR, the *bla*_ OXA-48_ , *bla*_ OXA-23_ and *bla*_ NDM_ specific primers produced the extended amplified DNA band in all MBLs strains. Fig. 2. shows the specific amplification band belonging to *bla*_ OXA-48_ , *bla*_ OXA-23_ and *bla*_ NDM_ genes. There were no any non-specific amplification band in electerophoresis (Fig. 1) and multiplex PCR was successfully optimized for rapid and simultaneous detection of three MBLs gene in *A. baumannii* strains.

## DISCUSSION

*Acinetobacter baumannii* is an important nosocomial infection agent responsible for serious infections (20). Among *A. baumannii* strains, metal-lo-β-lactamases (MBLs) producers are widespread worldwide and plasmid mediated MBLs are responsible for carbapenem resistance (21). Recently, frequency of MBLs producing *A. baumannii* strains are increasing and detection of MBLs producers are important in clinical settings (22). In addition, for epidemiologic surveys, multiplex-PCR technique may be very helpful and reduce the cost of various PCR reactions.

In this study, we used a set specific primers for rapid and simultaneously detection of MBLs genes including *bla*_ OXA-48_ , *bla*_ OXA-23_ and *bla*_ NDM_ . According to the results, multiplex PCR method could detect MBLs gene including *bla*_ OXA-48_ , *bla*_ OXA-23_ and *bla*_ NDM_ genes successfully. Non-specific amplification was not observed and we considered multiplex PCR assay as a specific method for detection of MBLs genes. Optimization of annealing temperature is very important in multiplex PCR. In this study the annealing temperature was optimized at 57 °C for simultaneously detection of MBLs genes.

There are several studies about detection of MBLs gene in *A. baumannii* strains by PCR but there are no study about simultaneously detection of *bla*_ OXA-48_ , *bla*_ OXA-23_ and *bla*_ NDM_ genes. Due to high prevalence of *bla*_ OXA-48_ and *bla*_ OXA-23_ genes among *Acinetobacter* spp. in Iran and because of few studies for detection of *bla*_ NDM_ , we used a suitable set of primers for simultaneously detection of mentioned gene using multiplex PCR for monitoring the spread these genes in Iran.

Poirel et al. designed a multiplex PCR using sets of 11 primers which targeted *bla*_ IMP_ , *bla*_ VIM_ , *bla*_ NDM_ , *bla*_ SPM_ , *bla*_ AIM_ , *bla*_ DIM_ , *bla*_ GIM_ , *bla*_ SIM_ , *bla*_ KPC_ , *bla*_ BIC_ and *bla*_ OXA-48_ genes (23). They reported a rapid and reliable technique for detection of carbapenemase genes in different clinical isolates and concluded that multiplex-PCR can provide a convenient tool for better evaluation of real prevalence of carbapenemase genes.

Aksoy et al. evaluated two multiplex PCR assays for simultaneous detection of MBLs gene including *bla*_ IMP-like_ , *bla*_ VIM-like_ , *bla*_ SIM-1_ and *bla*_ OXA-23_ , *bla*_ OXA-51_ and *bla*_ OXA-40_ and *bla*_ OXA-58_ . Their results indicated that the multiplex PCR was a potentially valuable tool for rapid detection of MBLs genes (24). Mostachio et al. used a multiplex PCR assay for simultaneous detection of carbapenem-resistant *Acinetobacter* spp. They showed that *bla*_ OXA-23-like_ was the most frequent carbapenemase identified among carbapenem-resistant *A. baumannii* strains recovered from four Brazilian hospitals (25).

Woodford et al. designed a novel multiplex-PCR assay for detection and distinguish the OXA carbapenemase genes including OXA-23 and OXA-58 like in *Acinetobacter* spp. and the results of this study showed that this assay can be used for monitoring of mechanisms responsible for carbapenem resistance in *Acinetobacter* spp. (26). Mlynarcik et al. developed a new set of primers for simultaneously detection of 11 genes including *bla*_ KPC_ , *bla*_ OXA_ , *bla*_ VIM_ , *bla*_ NDM_ , *bla*_ IMP_ , *bla*_ SME_ , *bla*_ IMI_ , *bla*_ GES_ , *bla*_ GIM_ , *bla*_ DIM_ and *bla*_ CMY_ . The results of that study revealed their technique was a reliable method for screening of all mentioned carbapenemase genes in *Enterobacteriaceae* (27).

Kamolvit et al. reported a multiplex PCR for detection of oxacillinase genes in *Acinetobacter* spp. In this study, the primers were designed to selectively amplify *bla*_ OXA-134-like_ , *bla*_ OXA-211like_ , *bla*_ OXA-213-like_ , *bla*_ OXA-214-like_ and *bla*_ OXA-228-like_ genes. These authors suggested the multiplex PCR specifically detect five different *bla*_ OXA_ subgroups and this method has potential to monitor the spread of these genes in *Acinetobacter* spp. (28).

The multiplex PCR assay showed to be specific for rapid detection of the three MBLs genes tested. No false positive and negative results was occurred during the assay indicating that target loci used in the study were specific for MBLs genes.

Our results also revealed that the multiplex PCR using three primers sets was able to detect simultaneously MBLs genes in a single reaction by the combinations of the different-size amplicons without any cross-reactivity.

## CONCLUSION

In this study we developed a multiplex PCR for rapid and simultaneous detection of three metallo-beta-lactamase genes including *bla*_ OXA-48_ , *bla*_ OXA-23_ and *bla*_ NDM_ in single reaction. Based on results, it can be concluded the multiplex assay is a useful tool for the rapid detection of genes encoding MBLs and could help in the implementation of measures for the control of the dissemination of carbapenem resistance in the hospital setting.
